# Boron-Incorporating Silicon Nanocrystals Embedded in SiO_2_: Absence of Free Carriers vs. B-Induced Defects

**DOI:** 10.1038/s41598-017-08814-0

**Published:** 2017-08-21

**Authors:** Daniel Hiller, Julian López-Vidrier, Sebastian Gutsch, Margit Zacharias, Michael Wahl, Wolfgang Bock, Alexander Brodyanski, Michael Kopnarski, Keita Nomoto, Jan Valenta, Dirk König

**Affiliations:** 1 Laboratory for Nanotechnology, Departament of Microsystems Engineering (IMTEK), University of Freiburg, Freiburg, Germany; 2 Institute for Surface and Thin Film Analysis GmbH (IFOS), Kaiserslautern, Germany; 3 The University of Sydney, Faculty of Engineering and Information Technologies, School of Aerospace, Mechanical and Mechatronic Engineering, Sydney, Australia; 4 Departement of Chemical Physics and Optics, Faculty of Mathematics and Physics, Charles University, Prague, Czech Republic; 5 Integrated Materials Design Centre (IMDC), UNSW, Sydney, Australia

## Abstract

Boron (B) doping of silicon nanocrystals requires the incorporation of a B-atom on a lattice site of the quantum dot and its ionization at room temperature. In case of successful B-doping the majority carriers (holes) should quench the photoluminescence of Si nanocrystals via non-radiative Auger recombination. In addition, the holes should allow for a non-transient electrical current. However, on the bottom end of the nanoscale, both substitutional incorporation and ionization are subject to significant increase in their respective energies due to confinement and size effects. Nevertheless, successful B-doping of Si nanocrystals was reported for certain structural conditions. Here, we investigate B-doping for small, well-dispersed Si nanocrystals with low and moderate B-concentrations. While small amounts of B-atoms are incorporated into these nanocrystals, they hardly affect their optical or electrical properties. If the B-concentration exceeds ~1 at%, the luminescence quantum yield is significantly quenched, whereas electrical measurements do not reveal free carriers. This observation suggests a photoluminescence quenching mechanism based on B-induced defect states. By means of density functional theory calculations, we prove that B creates multiple states in the bandgap of Si and SiO_2_. We conclude that non-percolated ultra-small Si nanocrystals cannot be efficiently B-doped.

## Introduction

In the first report about B-doping of Si nanocrystals (Si NCs) a quenching of the photoluminescence (PL) was observed and tentatively ascribed to Auger recombination^1^, i.e., non-radiative recombination of the photo-excited exciton with the B-induced hole. This explanation implicates that B-doping in ultra-small Si nanostructures works in the same way as in bulk-Si. However, from the bare observation of decreasing PL with increasing B-concentration, the alternative explanation based on B-induced non-radiative defects either in the Si NCs, or on its surface, or in the surrounding SiO_2_ cannot be excluded. While the Auger recombination concept is frequently discussed^1–7^, B-induced defects quenching the PL are less frequently mentioned^4, 8, 9^, though very likely to occur for the typically high B-concentrations used. Typical samples contain some 10^18^ NCs per cm^3^ and a certain fraction of the B-atoms will always remain in the SiO_2_ matrix surrounding the NCs. If on average 1 B-atom per NC shall be introduced in the sample, B-concentrations on the order of ~10^19^ cm^−3^ (equivalent to roughly 0.1 at%) are required and can be considered as low doping. Hence, B-concentrations in the range of ~10^20^ cm^−3^ (~1 at%) represent a comparatively high doping level that approaches the solubility limit of B in bulk Si (2 × 10^20^ cm^−3^ at 1100 °C)^10^. It is noted that impurity concentrations in the at% range are not necessarily dopants in the classical sense of the word, i.e., well-dispersed impurity atoms that either reside on substitutional or interstitial lattice sites. Impurities at that concentration level can induce the formation of Si-B alloys (e.g. silicon boride^11^) or ternary Si-B-O compounds, which involves the transformation of the host material and its structural properties. A prominent recent example for such fundamental changes are localized surface plasmon resonances found for ~7.5 nm Si NCs *hyperdoped* with up to 60% B and with measured free carrier densities in the mid-10^20^ cm^−3^ range (i.e., ~100 free carriers per NC)^12–14^. It cannot be excluded that a new binary Si-B phase is present here that exhibits metallic behaviour. We also want to point out that superconductivity was reported for Si doped with B in excess of 5 at%^15^. Apart from these extreme regimes, we argue that optical and electrical effects of B-doping should be visible from the low concentration level on, because there is no reason to assume a B-threshold concentration. Indeed, various optically and electrically measured results seem to indicate successful B-doping for lowly doped (<1 at%) Si NC samples^7, 16–18^. However, having a closer look at the respective fabrication parameters, it turns out that all these samples were grown with SiO_x_-stoichiometries beyond the percolation threshold (x ≈ 0.85 for thick films^19^, x ≈ 0.6 for very thin films in superlattice (SL) configuration^20^). Structurally, such samples do not consist of individual, spherical, and well-separated Si NCs, but of percolated nano-Si networks as shown in refs. 20–22. Accordingly, the constraints from confinement and nano-size effects are relaxed and free-carrier generation by B is more likely to occur, though the inferred B-doping efficiencies are very small (<5%^7^ and <2%^17^). If, however, Si NCs are regarded as a model system to investigate B-doping of ultra-small Si-nanovolumes, spherical NCs spatially isolated by SiO_2_ are mandatory. Here, we study experimentally and theoretically the properties of size-controlled and non-percolated B-incorporating Si NCs in SiO_2_ with the goal to disentangle the effects of PL quenching and free-carrier generation.

## Results and Discussion

### Incorporation of B in Si nanocrystals

**Figure 1:**
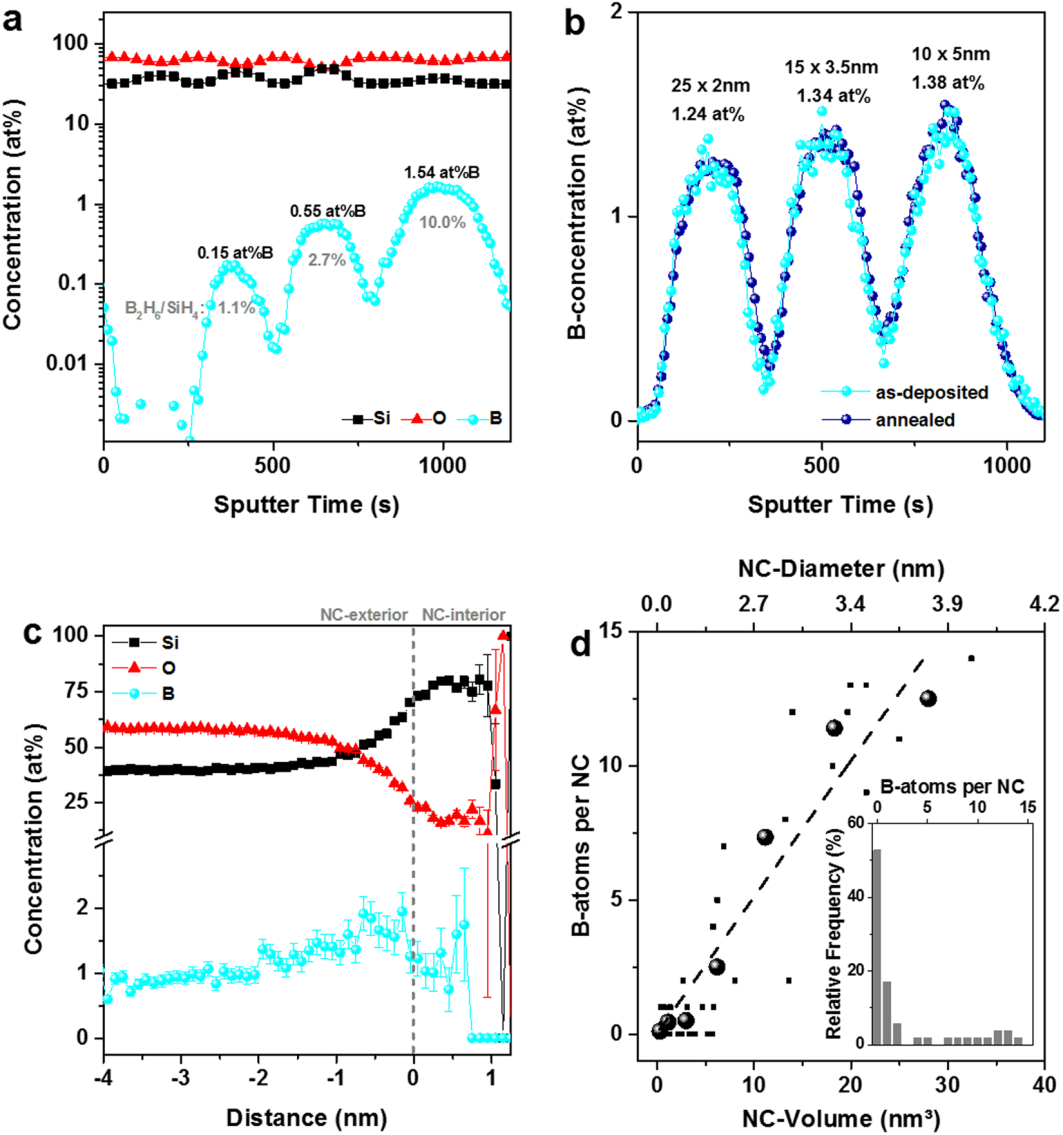
Analyses of B-concentration and B-incorporation. (**a**) Quantitative composition from MCs^+^-SIMS measurements of 4 SRO:B films (each ~50 nm) with different B_2_H_6_ gas flows, separated by SiO_2_ spacer layers. The B_2_H_6_/SiH_4_ gas flow ratio as well as the effective B-concentrations are given in numbers. (**b**) B-concentration of nominally identical ~50 nm SRO:B films with maximum B_2_H_6_ flux, which were deposited by consecutive ultra-thin films to demonstrate the small influence (approx. −14% relative) of transient deposition effects. In order to account for this effect, we redefine the nominal B-concentrations listed in (**a**) to: [B_min_] = 0.13 at%, [B_med_] = 0.47 at%, and [B_max_] = 1.32 at%. The other curve (dark blue) shows the same sample after 1100 °C-annealing. (**c**) Proxigram from the APT-measurement of a Si NC superlattice sample with 4.5 nm SRO:B_max_. The local B-concentration increases slightly from the faraway-matrix towards the NC-interface (negative distances). Inside of the NCs a significant B-concentration is detected in the near-surface region before it drops to zero towards the core of the NCs (positive distances). (**d**) Distribution of the number of B-atoms over the NC-size (small data points: APT raw data; large data points: averaged values from grouping the NCs into volume classes; dashed line: linear fit). The inset shows the relative frequency of NCs with $n$ B-atoms in their volume. Most notably, more than 50% of all NCs are B-free despite 1.32 at% B in the SRO.

Si NCs embedded in SiO_2_ are fabricated by annealing (1100 °C, 1 h) B-doped Si-rich oxide (SRO:B)/SiO_2_ SLs deposited by plasma-enhanced chemical vapour deposition (PECVD). Small amounts of B_2_H_6_ are added to the SiH_4_, O_2_, Ar gas mixture to introduce B in SRO, while the SiO_2_ barrier layers are not doped. In order to determine the initial B-concentration, we use molecular Cs^+^ secondary ion mass spectrometry (MCs^+^-SIMS)^23^. As shown in Fig. 1a we achieve B-concentrations in SRO in the range of 0.15 to 1.54 at%. Besides, the Si- and O-concentration is not completely unaffected by adding B_2_H_6_, though all SiO_x_-stoichiometries remain in the x ≈ 1.0 ± 0.2 range, which is well below the percolation threshold. Hao *et al*. argued for their sputtered samples that a reduced Si-content occurs due to additional oxygen from the B_2_O_3_ target^4^, which is certainly not the explanation for the PECVD process used here. However, the hydrogen from B_2_H_6_ might influence the plasma chemistry and thereby the Si/O ratio. On the other hand, transient effects during the ultra-low rate deposition (<1 Å/s) might occur that change the B-concentration. Figure 1b depicts the B-concentration in 50 nm SRO:B films that were not deposited in one step but from sequential thin films as used in the actual SRO:B/SiO_2_ SLs. We observe a slightly lower B-concentration compared to the thick one-step film with maximum B_2_H_6_ flux (1.54 at%, Fig. 1a): 1.24 at% for 25 × 2 nm, 1.34 at% for 15 × 3.5 nm, 1.38 at% for 10 × 5 nm. On average, the transient deposition effects reduce the B-concentration in SLs to ~86% compared to the thick one-step film. In the following, we will therefore refer to the nominal B-concentrations as [B_min_] = 0.13 at%, [B_med_] = 0.47 at%, and [B_max_] = 1.32 at%. The second data set in Fig. 1b shows the B-concentration of the same SRO:B stack after annealing at 1100 °C. Both curves are hardly distinguishable, i.e., the MCs^+^-SIMS-measurement is not influenced by the structural changes the SRO undergoes during annealing and the B-concentration does not change during annealing. The latter is in agreement with the very low diffusion coefficient of B in SiO_2_^4^.

Whereas SIMS can quantify the B-concentration in the SRO from which the Si NCs are formed by annealing, we use atom probe tomography (APT) to detect individual B-atoms with sub-nm spatial resolution in the Si NC/SiO_2_ system. In Fig. 1c the proxigram (proximity histogram^24^) of a Si NC SL sample with 4.5 nm SRO:B_max_ using 60% Si iso-concentration surfaces is shown. In contrast to typical proxigrams for P-doped Si NCs^22, 25, 26^ we do not observe a strong pile-up of dopants exactly at the Si/SiO_2_ interface and a stable considerable level of dopants in the NC-volume. It was shown before for sputtered B-doped Si NC samples^22, 25^ that the proxigram exhibits a constant B-level in the SiO_2_ matrix and a concentration-drop towards the inner NC volume. Here, we observe a slightly increasing B-concentration in the oxide matrix towards the NC-interface and a considerable B incorporation in the near-surface volume of the NC-interior, but almost no boron in the NC-cores. This behaviour is consistent with the segregation coefficient of ~0.3 for B in bulk-Si/SiO_2_^27^, which is based on the lower solubility of B in Si than in SiO_2_. On the other hand, nanocrystals are subject to self-purification^28^, so that the reason for the evanescent B-concentrations in the NC-core might not only be related to insufficient solubility. In this picture, the small B-concentration increase in the Si/SiO_2_ interface region could be the result of two counter-acting forces during annealing: Self-purification tries to eject B-atoms from the NC into the matrix but the solubility limit of B in SiO_2_ is reached for values at around 1 at% and the only region left to deposit B is the interfacial SiO_x_ transition shell^29, 30^. Furthermore, it was theoretically calculated that B in silanol (OH) terminated Si NCs has an energetically most favourable site just a few Å under the surface^31^. For B in fully SiO_2_-embedded Si NCs theoretical results even suggest an energetical preference for real surface incorporation^32^. Besides, our samples represent a heterogeneous semiconductor-dielectric system with inherently different evaporation field strengths. At a given field the Si precipitates evaporate faster than the surrounding SiO_2_ which causes local magnification effects (LME)^33, 34^ that project some of the atoms in the exterior of the NCs towards the interior. Accordingly, we measure 15–20 at% of oxygen in the Si NC-volume. The B-concentration is also subject to this projection artefact, so that the true values in the near-surface region of the NC-volume will be lower than shown in Fig. 1c. The last factor that influences the measurement is the limited detection efficiency of APT (here 57%). To some extent, both effects compensate each other and in absence of any measurement technology with higher precision, we stick in the following to the as-measured data. In Fig. 1d the amount of B-atoms per NC as function of NC-size is shown. As observed before for P-dopants^35^ there is a clear trend for larger NCs to incorporate more B-atoms, as indicated by the linear fit (dashed line). The inset of Fig. 1d shows the frequency of NCs that incorporate $n$ B-atoms. Interestingly, more than 50% of the NCs in the sample grown from SRO:B_max_ with 1.32 at% B do not contain a single B-atom. Out of the NCs that incorporate one or more B-atoms, ~90% have B-concentrations above the solubility limit, i.e., they are B-supersaturated, which increases the probability for interstitial rather than substitutional B-incorporation. In contrast, less than 20% of P-incorporating NCs exceed the solubility limit^35, 36^.

Altogether, the APT analysis of the sample doped with the highest B_2_H_6_ flux allows to conclude that only half of the NCs actually incorporate B-atoms and, according to the proxigram, the B-atoms are located very close to the surface of the NCs. This observation constitutes for boron a high probability to be not fully Si-coordinated but partially connected to O in the SiO_x_ transition shell. A significantly higher exothermal formation energy of the B–O bond over the B–Si bond^37^ corroborates this statement. By definition, B-atoms in this configuration cannot become dopants. Furthermore, a near-surface location of the dopant is the worst scenario in terms of dopant charge screening within the semiconductor, which gives rise to enhanced dielectric confinement and high ionization energies^38^.

**Figure 2:**
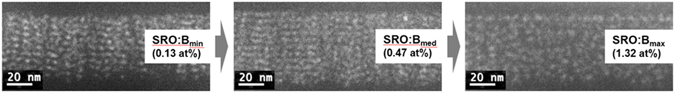
Influence of B on the SiO_2_ viscosity. EFTEM cross-section images of 1100 °C-annealed 10-bilayer 4-nm-SRO:B/2-nm-SiO_2_ SLs, filtered around the Si-plasmon loss energy. The layered structure is well preserved for the lowest and the medium B-concentration but the NC-stacking order is less clearly pronounced for the highest B-concentration. Obviously, the threshold for SL-preservation and thereby for NC size-control is slightly above 1 at% B.

Boron is known to reduce the viscosity of SiO_2_ at high temperatures. Therefore, the structural integrity of the SL stacking order during annealing cannot be taken for granted. Energy-filtered transmission electron microscopy (EFTEM) cross-section images, as shown in Fig. 2, demonstrate that the SL structure is affected by high B-concentrations: The layer-wise arrangement of NCs diminishes for concentrations >1 at% B. This is related to the lower viscosity of SiO_2_:B during the annealing (1100 °C), i.e., the SiO_2_ barrier layers which separate the NC-layers are softened by B. For comparison, borosilicate glasses (8–13% B_2_O_3_) are well known to soften already at ~820 °C. It can be anticipated from the EFTEM images that B-concentrations ≫1 at% cannot be used without losing NC-size control mediated by the SL. Hence, the insufficient B-incorporation into NCs (cf. Fig. 1d) cannot be improved by adding increasing amounts of B, if at the same time small NC sizes and size distributions are to be maintained.

### Luminescence of B-incorporating Si NCs

The PL-intensity of Si NCs with boron is typically reported to be lower than for undoped reference samples, even for very small B-concentrations of ≤0.1 at%^1–5, 7, 8^. So far, only Puthen-Veettil *et al*. observed a ~40% PL-intensity increase for their lowest B-sputter target power, accompanied by a decreased dangling bond (DB) defect signal measured by electron spin resonance^6^. This behaviour is typical for phosphorus dopants (cf. ref. 39 and references therein) and generally attributed to DB-defect passivation by P. In the case of boron, this mechanism might be present as well.

**Figure 3:**
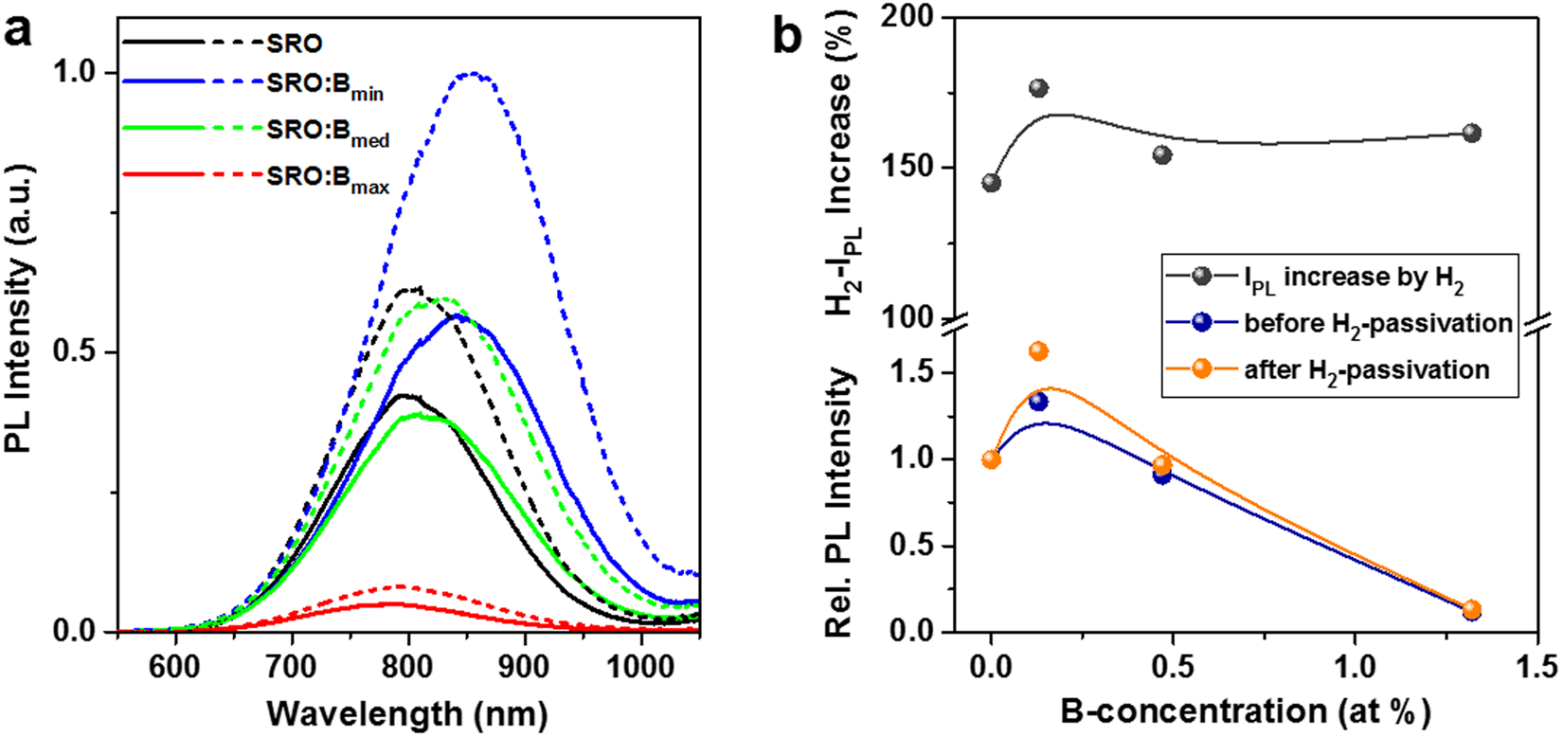
Photoluminescence of B-incorporating Si NCs. (**a**) PL spectra of NC samples before (solid lines) and after (dashed lines) H_2_-passivation with nominal B-concentrations of: [B_min_] = 0.13 at%, [B_med_] = 0.47 at%, and [B_max_] = 1.32 at%. While all samples have similar peak positions, the highest B-concentration leads to a strong luminescence quenching by one order of magnitude. (**b**) The PL peak analysis demonstrates the small PL-intensity gain by 0.13 at% B, irrespective of H_2_-passivation and similar intensities of the 0.47 at% samples compared to the undoped references. The H_2_-passivation increases the PL-intensity on average by ~60%. All lines (splines) are just a guide to the eye.

In Fig. 3a the PL spectra of the Si NC samples with varying B-concentrations are shown together with the same sample set passivated at 450 °C for 1 h in pure H_2_. Boron does not significantly influence the PL peak positions and we attribute the differences, at least partially, to the small changes in the excess-Si content of the samples (cf. Fig. 1a). All H_2_-passivated samples are slightly red-shifted in their PL by 10–15 nm, which is related to the well-known preferential emission enhancement of larger NCs within the ensemble (larger surface area implicates higher probability to have a non-radiative Si/SiO_2_ interface defect)^40–42^. As shown in the peak analysis in Fig. 3b, the PL-increase by H_2_ is on average around 60%, irrespective of B-doping. It is evident on first sight, that small and medium B-concentrations have only little impact on the PL-intensity, while the PL of the B_max_-samples is quenched by a factor of 10. Like in ref. 6, we observe a 30–60% increase in PL-intensity for the lowest B-concentration B_min_. Interestingly, the B_med_-samples have almost the same PL-intensity as the undoped reference samples, i.e., for 0.47 at% B the PL-enhancing and the PL-quenching effect seem to compensate each other. This is a surprising result since a simple linear extrapolation of the APT data measured for the B_max_-sample reveals that the B_med_-sample contains at least $\sim 1/3$ B-incorporating NCs. In case the B-atoms do provide free carriers, a significant loss of PL-intensity should be visible when only $\sim 2/3$ PL-active NCs are left. In addition, the PL-peak should blue-shift since the largest NCs within each sample have the highest B-concentrations (cf. Fig. 1d). Instead, we observe very similar peaks for B_med_ and the B-free reference, which is hardly explainable by active B-dopants. Furthermore, the sudden drop in PL-intensity when increasing the B-concentration from 0.47 to 1.32 at% (B_med_ to B_max_) indicates something like a B-threshold that is required to activate the B-induced PL-quenching – a parameter incompatible with conventional B-doping. We derived above (cf. inset of Fig. 1d) that roughly half of the NCs in the B_max_ sample are B-free and therefore potentially PL-active. The circumstance that this ~50% fraction emits only ~10% of the luminescence compared to the undoped sample is a clear indication for a B-induced non-radiative defect centre. Another argument against Auger recombination with B-induced free charge carriers arises from the inadequately small PL-blueshift of the B_max_-samples that does not reflect the 1 order of magnitude intensity quenching. We note that the nature of the B-induced defect centre cannot be a simple DB-type defect since the PL-quenching is nearly unaffected by H_2_-passivation.

**Figure 4:**
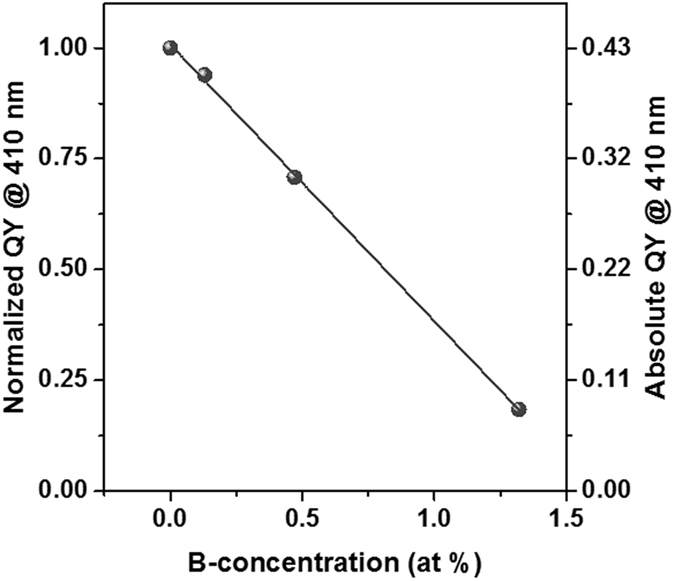
Luminescence Quantum Yield of B-incorporating Si NCs. The ratio of emitted photons (at PL peak) and absorbed photons (410 nm excitation) of H_2_-passivated, 4.5 nm Si NC samples decreases almost linearly with increasing B-concentration. The solid line is a linear fit with a slope of approximately −0.6, i.e., for NCs grown from SRO:B with 1 at% the QY is quenched by 60% relative compared to an intrinsic sample.

In order to exclude a major uncertainty of standard PL-spectroscopy, i.e., the sensitivity of the PL-intensity on the number of luminescent NCs and their absorption cross-section in each sample, we measured the luminescence quantum yield (QY)^43^. By measuring the ratio of emitted and absorbed photons, these ambiguities are ruled out and the true luminescence efficiency of each sample is revealed. The QY-data of H_2_-passivated NC samples as function of B-concentration is given in Fig. 4. The absolute QY value of up to ~43% for the undoped reference sample is (to our knowledge) the highest ever reported for matrix-embedded Si NCs. Recently, a record efficiency of 35% was published^44^, which is clearly outperformed here. Higher QY of 60–70% was only achieved for organically passivated, free-standing Si NCs^45^. Due to the low initial fraction of defective, dark NCs in our samples, we have a highly sensitive system to observe the influence of non-radiative centres introduced by B-doping. In direct comparison to Fig. 3, we see that the QY does not show the same trends like the PL-intensity. Neither the PL-intensity enhancing effect of 0.13 at% (B_min_) nor the almost identical PL peaks of the reference and the 0.47 at% (B_med_) samples (Fig. 3a, green and black lines) are reproduced in the QY. Only the strong quenching at 1.32 at% (B_max_) is clearly visible. It is likely that structural effects of B on the NC formation or crystallization and the slightly different excess-Si concentrations are the reason for the PL-behaviour observed above. We note here that, generally, luminescence QY and not just PL-intensity should be analysed to draw conclusions about the impact of impurities on Si NC samples since this method circumvents disturbing structural side effects. Whereas no direct APT-data is available about B-incorporation in NCs grown from low or medium B-doped SRO, we can presume that the fraction of B-incorporating NCs of such samples correlates to the small loss of QY. Therefore, we cannot identify the origin of QY-loss (Auger recombination with B-induced free carriers vs. B-induced non-radiative defects). On the other hand, the ~50% B-free NCs in the B_max_-sample emit PL with only 18% of the efficiency compared to the intrinsic reference sample. This is a clear indication that at least partially a defect-related luminescence quenching is present.

### Electrical properties of B-incorporating Si NCs

**Figure 5:**
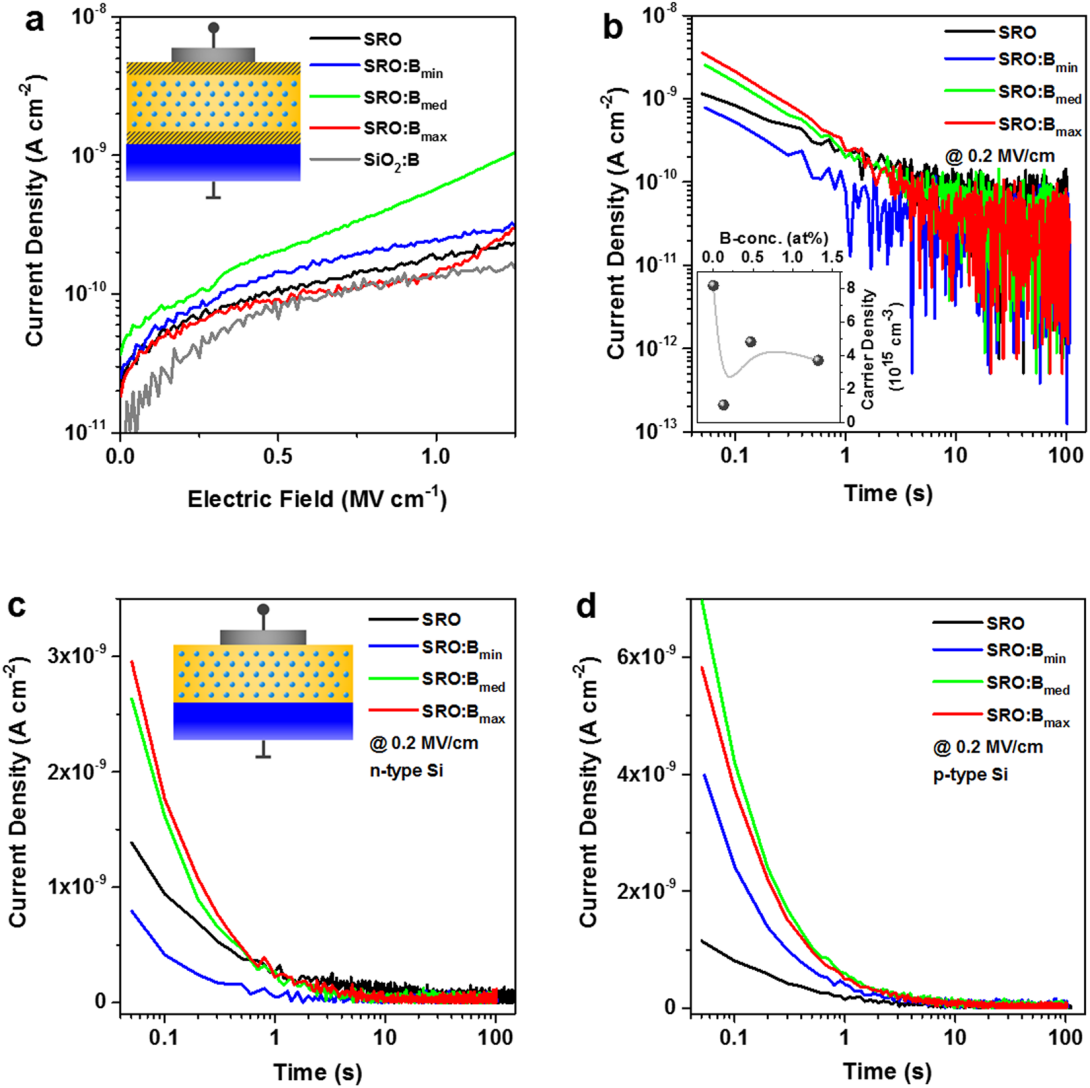
Electrical properties of B-incorporating Si NCs. (**a**) J–E data of B-doped Si NC superlattices and references configured as blocking-MOS capacitors (on *n*-type Si) that prevent carrier injection from gate or substrate. The inset shows a schematic sample cross-section. The displacement current curves show no J-peak, indicating the absence of substitutional B-atoms that undergo field ionization. (**b**) J-t measurements on the blocking-MOS capacitors at **E** = 0.2 MV/cm. The transient currents of B-doped and undoped samples are similar and the corresponding apparent carrier densities derived from integration (inset) do not show any effect of B-doping. (**c**,**d**) J-t measurements on MOS capacitors without thick injection barriers (cf. schematic in the inset of (**c**)) at E = 0.2 MV/cm on *n*-type Si substrate (**c**) and *p*-type Si substrate (**d**). In both cases, the polarity of the gate bias was chosen to achieve accumulation in the substrate. Under steady-state conditions (*t* ≥ 10 *s*) the B-doped Si NC samples do not show an increased conductivity for any charge carrier type.

Since the luminescence measurements done here cannot distinguish between non-radiative recombination with free carriers or defects, we investigate the current-voltage (I-V) properties of B-incorporating Si NCs in metal-oxide-semiconductor (MOS) capacitors. In the first place, only transient displacement currents are measured by means of NC-superlattice stacks sandwiched between 10 nm thick SiO_2_ injection blocking layers^46^. At low electric fields (**E**) this blocking-MOS device allows for the observation of a current peak, if dopant atoms are ionized by the **E**-field and release free carriers that subsequently accumulate under the gate blocking oxide^17, 35, 46^. Figure 5a shows the current densities (**J**) of the B-incorporating Si NC samples together with the intrinsic NC sample and a B-doped SiO_2_ (0.6 at%) thin film of equal thickness. All **J**-**E** curves are hardly distinguishable and there is no indication for a **J**-peak. The SiO_2_:B reference sample proves that the B-atoms in the silicon oxide matrix between the Si NCs does not contribute to any charge carrier generation. The slightly higher current density of the B_med_-sample might be again related to the marginally higher excess-Si content^47^.

In Fig. 5b the current density of a blocking-MOS capacitor as function of time at **E** = 0.2 MV/cm is plotted, i.e., at a typical **E**-field where P-dopants would cause up to two orders of magnitude higher **J** ^35, 46^. Here, we observe very similar **J**-t curves for all samples, irrespective of B-doping. After a few seconds, the transient displacement currents reach the noisy sub-100 pA cm^−2^ level where the internal charge redistribution and dielectric relaxation is completed. By integrating over time, the total amount of carriers is calculated (cf. refs. 17, 35 and 46), which typically reaches values on the order of magnitude of 10^17^ cm^−3^. As shown in the inset of Fig. 5b, our samples seem to have carrier densities in the 10^15^ cm^−3^ range with, oddly enough, the B-free reference having the highest value. However, given the very short time scale (≤1 second) on which the I-t curves differ significantly before reaching the noise level, indicates that just dielectric relaxation rather than charge redistribution takes place. We therefore deduce that the carrier densities (Fig. 5b inset) are just an artefact caused by dielectric relaxation and can thus be considered as an upper limit.

Finally, B-incorporating Si NC superlattices in normal MOS-capacitors, i.e., without thick SiO_2_ injection barriers, are investigated to find out whether the presence of B-dopants increases the charge carrier transport. The results for non-blocking MOS-capacitors in accumulation are shown in Fig. 5c for *n*-type and in Fig. 5d for *p*-type Si substrates. Apparently, neither increased electron- nor hole-conductivity is present for times ≥10 s, when the initial transient currents faded and steady-state conditions are reached. Once again this is in contrast to P-doped Si NCs^48^. We want to emphasise that little information can be derived from initial time interval at *t* < 1*s* where dielectric relaxation and internal charge redistribution takes place. Although the two samples with higher B-concentrations seem to have larger initial currents than the other two samples, there is no systematic trend with B-concentration and in particular no difference between electron and hole transport.

Given the absence of a **J**-peak (Fig. 5a) and enhanced current transport (Fig. 5c,d) for B-incorporating Si NC samples, we conclude that there is no significant amount of ionisable B-acceptors. Hence, there are no free carriers in the B-incorporating Si NCs from thermal ionization at room temperature. Furthermore, free carriers cannot be generated by field-ionization. The latter aspect deviates substantially from phosphorus, where the small fraction of NC-incorporated P-atoms that reside on a substitutional site does provide charge carriers, if ionized by an external electrical field^35^. Consequently, the few B-atoms incorporated in small (~4 nm) and non-percolated Si NCs are predominantly located on interstitial sites. We note that similar measurement on percolated NC-networks revealed a **J**-peak and thereby a small fraction of substitutional B-atoms is anticipated for larger nano-Si volumes^17^. As argued above, it is also possible that B-atoms in very small NCs resides very close to the surface, where they are not completely Si-coordinated or subject to a huge dielectric confinement^38^ due to the adjacent SiO_2_ matrix. The preferred near-surface position of B in Si NCs is in accord with APT-data (Fig. 1c and refs. 22 and 25) and theoretical calculations of H-terminated^9^, OH-terminated^31^ and SiO_2_-embedded Si NCs^32^. Concerning the B-ionization energy, which we cannot derive from our data (no **J**-peak), the work of Lechner *et al*. indicates for B-concentrations of 3 × 10^16^ cm^−3^ and 2 × 10^18^ cm^−3^ in ~20 nm free-standing Si NCs (equivalent to ~0.1 and ~8 B-atoms per NC) values of 420 and 280 meV, respectively^49^. Thus, even Si NCs with sizes too large for significant quantum confinement have considerable B-ionization energies, which underlines the crucial role of dielectric confinement, or respectively, the influence of hole-trapping DB-defects, as argued by the same authors.

### B-induced states in the Si NC/SiO_2_ system

**Figure 6:**
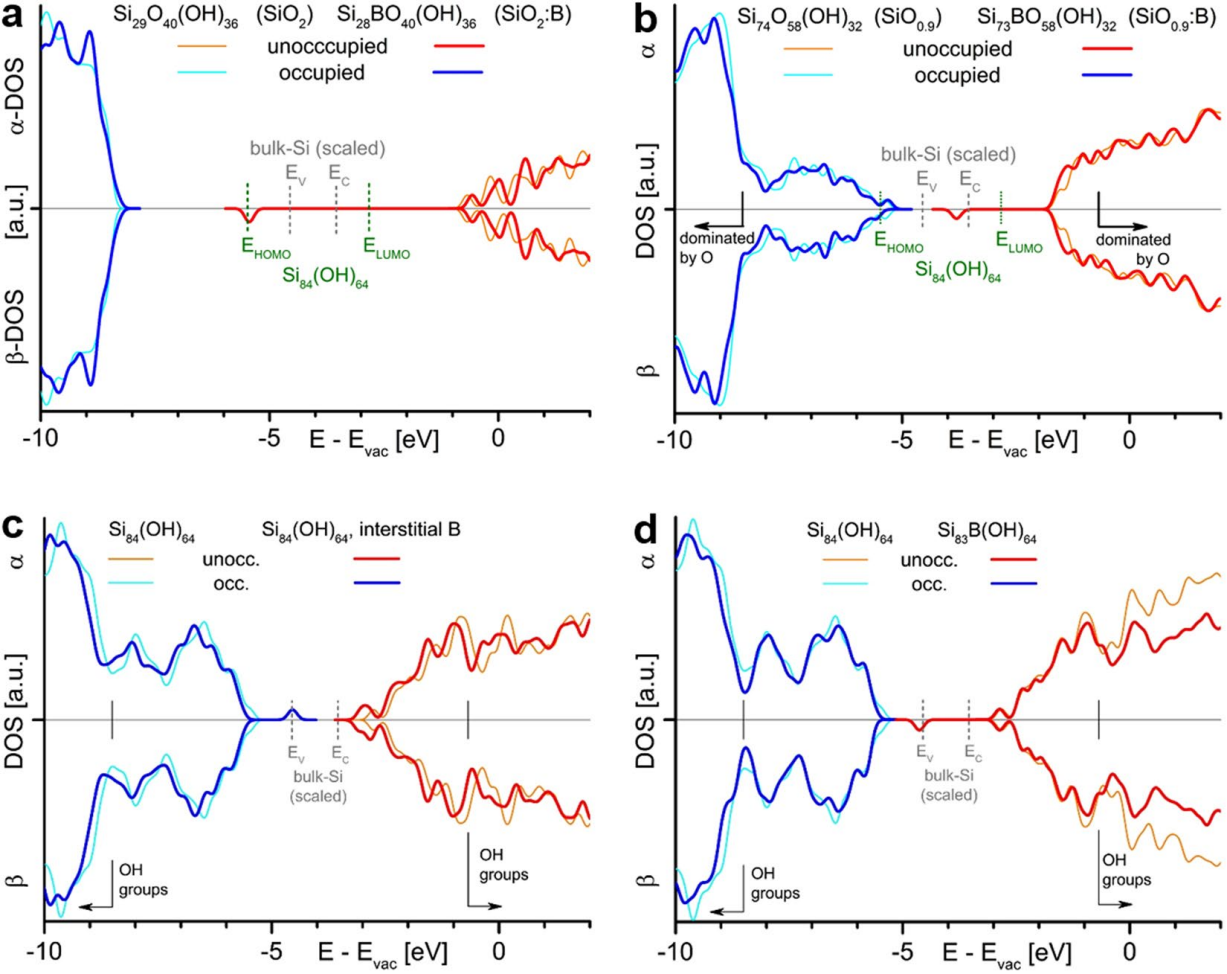
Results of density functional theory (DFT) calculations for B in the Si/SiO_2_ system. Density of states (DOS) of pure, intrinsic approximants (unoccupied states in orange and occupied states in cyan) and B-incorporating approximants (red and blue, respectively). (**a**) B in SiO_2_ creates an unoccupied state that is only in the energetic vicinity of the highest occupied states (HOS; valence band edge in bulk) for Si NCs ≤ 1.5 nm as indicated by the green dashed line^59–61^ but outside of the gap for larger NCs. (**b**) In the suboxide transition shell around the NCs (simulated here as SiO_0.9_) B introduces an unoccupied state that has almost mid-gap character, which makes it an efficient non-radiative recombination centre. (**c**) For a B-atom on an interstitial site in a Si_84_(OH)_64_ approximant (1.5 nm NC) an occupied state close the HOS (VBE in bulk) occurs, whereas for a substitutional B-atom in this approximant (**d**) an unoccupied state at the same energetic position occurs, as expected from conventional B-impurity doping.

As shown above, the majority of B-atoms reside in the SiO_2_ matrix or in the suboxide transition shell surrounding the NCs. This raises the question how boron in these configurations changes the electronic environment of the NCs. We use density functional theory (DFT) to calculate the density of states (DOS) of SiO_2_:B (Fig. 6a) and SiO_0.9_:B (Fig. 6b) together with the respective intrinsic approximants. The B-free oxides do not contain any states in or near the fundamental Si NC gap (as expected). However, for SiO_2_:B we observe a state that is only in the vicinity of the highest occupied states (HOS; equivalent to the valence band edge (VBE) in bulk) for very small NCs of 1.5 nm in diameter (Fig. 6a). For larger Si NCs this state is outside of the fundamental NC-gap. At the NC-interface, simulated here with SiO_0.9_:B, the unoccupied state shown in Fig. 6b is shifted to a position slightly above mid-gap for bulk and nanocrystalline Si. At this energetic position, it can efficiently capture the electron of a photo-excited exciton, which causes non-radiative recombination similar to a Si-DB defect.

For those B-atoms that are incorporated into the Si NC core, two configurations exist: interstitial and substitutional. In Fig. 6c the DOS of a 1.5 nm NC with an interstitial B-atom is shown to contain a B-induced occupied state near the VBE of bulk-Si. For any NC that is subject to bandgap widening by quantum confinement, this state is energetically located slightly above the HOS, so that its electron could immediately recombine with a photo-generated hole. Therefore, interstitial-B is also regarded as a PL-quenching centre. The DOS of the substitutional B-configuration is shown in Fig. 6d. There is an unoccupied state slightly above the HOS of a NC, which would make it a hole-generating acceptor state, if it is thermally ionisable and, moreover, if this configuration exists in sufficient concentrations. For the first condition, this state would have to be as close to the HOS as in the case of bulk-Si, i.e., ~45 meV. Otherwise, the exponential dependence of the ionization probability on the ionization energy decreases the free carrier density dramatically. The substitutional incorporation of a B-atom into the lattice of a small Si NC requires a rather high formation energies^31, 32, 50^ of beyond ~1 eV. This energy has to be provided by the 1100 °C-annealing that forms the NCs, which is equivalent to a thermal energy of only ~0.12 eV. Hence, substitutional B is very unlikely to occur in small, non-percolated NCs, which is in accord with the absence of any electrical activity (cf. Fig. 5). Although differences between the DOS shown here for boron and that calculated for phosphorus in the same approximants^51^ exist, the main conclusion is the same: Interstitial dopants and dopants in the suboxide transition shell are the most likely candidates to create defect states in the bandgap, while majority charge carrier generation from substitutional dopants is impeded by high ionization energies.

Whereas a number of B-induced states in the Si/SiO_2_ system with energetic vicinity to the fundamental NC-gap are present, we cannot unambiguously identify which B-induced state is the dominant PL-quencher. However, contributions of Auger recombination with B-induced free-carriers appear to be virtually nil.

## Conclusion

B-atoms were shown to be incorporated in small (~4 nm) non-percolated Si NCs, though at a lower concentration and with lower probability than e.g. P-dopants. In addition, B prefers a near-surface location in Si NCs. PL-QY measurements reveal that B quenches the luminescence of NCs with a nearly linear dependence on the B-concentration. In contrast, no free-carriers are measured electrically and even field-ionization is not capable to generate significant amounts of carriers. In other words, B-doping has no measurable impact on the I-V and I-t behaviour of Si NC/SiO_2_ MOS capacitors. We conclude that the vast majority of B-atoms are incorporated on interstitial lattice sites, which is supported by its high substitutional formation energy. The electrical inactivity of B in small NCs and the apparent absence of B-atoms on Si lattice sites render B-induced defect states to be the main origin of luminescence quenching. DFT results suggest that especially interstitial-B and B in the suboxide transition shell surrounding the NCs act as non-radiative centres.

The absence of successful B-doping, as shown here, is restricted to small and individual, i.e. non-percolated, Si NCs. It was shown in the literature, that percolated nano-Si networks do exhibit some electrical activity^17^, which supports the presence of some field-ionisable, substitutional B-atoms in such samples. However, considering Si NCs as a model system to study B-doping in the limit of few-nm Si-nanovolumes, spatially-isolated and mainly spherical structures are mandatory. Another restriction for this model system arises from the B-concentration, which has to remain within the doping-range (~1 at% at max) and must not exceed the semiconductor-metal transition threshold (quasi-metallic properties such as plasmon resonances) or the threshold for Si-B alloy formation. Specifically, for size- and shape-controlled Si NCs the fabrication via SiO_x_/SiO_2_ superlattices is a convenient approach, unless exceeding B-concentrations deteriorate the stacking order via a reduction in viscosity of SiO_2_:B (as shown by EFTEM for ≥1.3 at% B). All these factors implicate that there is no technological solution to enable B to become an efficient impurity dopant for ultra-small Si nanovolumes. An alternative approach to facilitate *p*-type behaviour in such structures is the generation of Al-induced acceptor states in Si-adjacent SiO_2_ that capture electrons from the Si valence band, which leaves holes as free carriers behind^52^.

## Methods

### Sample fabrication

Si NC/SiO_2_ superlattices were fabricated by PECVD (using SiH_4_, O_2_ and Ar^53^) of alternating 4.5 nm SiO_x≈1.0_ and SiO_2_ barrier layers on (100)-oriented Si wafers. For B-doping, 0.18–0.94 sccm of 10%-B_2_H_6_/SiH_4_ were added, depending on the intended B-concentration. The samples for APT had 5 nm SiO_2_ barriers and 30 bilayers, those for PL and PL-QY 4 nm barriers and 20 or 40 bilayers, respectively. All samples dedicated to I-V measurements had total thicknesses of ~100 nm with 2 nm SiO_2_ barriers and 14 bilayers (injection-blocking MOS devices with 10 nm thick SiO_2_ buffer and capping layers) or 17 bilayers (non-blocking MOS-capacitors), respectively. The substrates of the I-V samples were either *n*-type (P, 1–30 Ω cm) or *p*-type (B, 1–30 Ω cm). After deposition, all samples were annealed in a quartz glass tube furnace at 1100 °C for 1 h in high-purity N_2_ and subsequently defect passivated at 450 °C for 1 h in pure H_2_. In order to fabricate MOS-capacitors for electrical characterization, aluminium contacts were thermally evaporated and photolithographically structured.

### Sample characterization

MCs^+^-SIMS was measured using a Cameca IMS-4f with 3 keV Cs^+^ ions. APT was measured with a Cameca-LEAP™ 4000X Si with a pulsed UV laser (355 nm, 100 pJ, 250 kHz), a cooled specimen holder (~40 K) and a chamber pressure of 10^−12^–10^−11^ Torr. For data reconstruction IVAS™ (version 3.6.6) was used. APT specimen (i.e., needle-shaped tips attached onto the apex of a Mo support grid) were fabricated using a Zeiss Auriga focused ion beam scanning electron microscope (FIB-SEM). For EFTEM imaging a JEOL 2010 operated at 200 kV and equipped with a Gatan imaging filter (GIF-863 Tridiem) was used. The energy filter was set to 16 eV (Si plasmon loss) with a 3.5 eV window. PL was carried out using a LN_2_-cooled CCD camera attached to a single grating monochromator with excitation by a HeCd laser (325 nm, ~3 mW/cm²). The luminescence QY was determined on samples deposited on quartz glass and a setup based on an integrating sphere, for details see ref. 43. I-V and I-t were measured in accumulation using an Agilent B1500A semiconductor device analyser. The MOS-capacitors were contacted by W-needles in a Cascade M150 Prober located in a shielded dark box.

### Hybrid density functional theory (h-DFT) calculations

Calculations were carried out in real space with a molecular orbital basis set (MO-BS) and the h-DFT method described below, employing the Gaussian03 and Gaussian09 program packages^54, 55^. Initially, the MO-BS wavefunction ensemble was tested and optimized for stability with respect to describing the energy minimum of the system (variational principle; stable = opt) with the B3LYP hybrid DF method^56, 57^ using a 6–31 G(d) MO-BS^58^ (B3LYP/6-31 G(d)). This MO wavefunction ensemble was then used for the structural optimisation of the approximant to arrive at its most stable configuration (maximum integral over all bond energies), again following the B3LYP/6-31 G(d) route. Using these optimized geometries, their electronic structure was calculated again by testing and optimizing the MO-BS wavefunction ensemble with the B3LYP/6-31 G(d) route. Root mean square (RMS) and peak force convergence limits were 8 meV/Å and 12 meV/Å, respectively. Tight convergence criteria were applied to the self-consistent field routine. During all calculations, no symmetry constraints were applied to MOs. Further accuracy evaluations can be found elsewhere^59, 60^. Electronic DOS were calculated from MO eigenenergies, applying a Gaussian broadening of 0.2 eV.
